# Prevalence of Khat chewing in college and secondary (high) school students of Jazan region, Saudi Arabia

**DOI:** 10.1186/1477-7517-6-11

**Published:** 2009-06-20

**Authors:** Hussein M Ageely

**Affiliations:** 1 Department of internal medicine, Jazan University, P O Box 1289, Jazan 45142, Kingdom of Saudi Arabia

## Abstract

**Background:**

Khat is widely consumed among the youth of Jazan region of Saudi Arabia. However, its prevalence is not well documented.

**Objective:**

This study was conducted to assess the prevalence and associated risk factors of khat chewing among college and secondary school students in Jazan region.

**Methods:**

The study was conducted in May 2006 in the colleges and secondary schools in Jazan region. A sample of 10,000 students aged between 15 and 25 years was randomly selected. Students in each year of study were selected by systematic random sampling technique. Self-administered questionnaire was used for data collection.

**Results:**

The overall prevalence of khat chewing in all the studied population was 21.4% (colleges 15.2% versus schools 21.5%). There were 3.8% female khat chewers and 37.70% male Khat chewers. Significant differences were found between khat chewers according to age, gender and residence (p < 0.05). The prevalence was different in different colleges and in different provinces of Jazan region.

**Conclusion:**

The prevalence of Khat chewing seems to be high among male students and not remarkable among female students. The use of Khat is significantly associated with age, gender, residence and school and college education (p < 0.05) among students of Jazan region. Strong measures need to be taken for greater awareness among school and college students to reduce its prevalence.

## Background

Khat is a natural stimulant from the Catha Edulis plant that is cultivated in the Republic of Yemen and most of the countries of East Africa. Its young buds and tender leaves are chewed to attain a state of euphoria and stimulation [1]. The khat chewers experience a sense of increasedenergy levels, increased alertness and ability to concentrate, improvement in self-esteem and an increase in libido [2].

There is fairly extensive literature on the potential adverse effects of habitual use of khat on mental, physical and social well-being [3]. Some khat chewers experience anxiety, tension, restlessness, hypnologic hallucinations, hypomania and aggressive behaviour or psychosis [4,5]. Chronic consumption can lead to impairment of mental health, possibly contributing to personality disorders and mental deterioration [6,7]. Khat leaves has vasoconstrictor properties [8] that may lead to elevated blood pressure, increases in heart rate and increased incidence of acute myocardial infarction (AMI) [9,10]. Gastro-intestinal hazards include constipation, stomatitis, esophagitis and gastritis [11]. A significant association between the habit of khat chewing and the development of haemorrhoidal disease was reported [12]. Besides damaging health, Khat has adverse socio-economic consequences effects on many other aspects of life including the loss of thousands of acres of arable land and billions of hours of work [13].

With the increasing evidence of the harmful effect of khat on the general health and the social problems associated with its use, the level of prevalence of khat among the population and its associated risk factors is important. Several reports showed that the prevalence of khat use differs according to age, gender, residence and occupation [14-19]. A survey carried out in a rural Ethiopian community [16] found that the prevalence of current khat use was 50%. A study performed in three towns in south-western Uganda [18] showed that the use of khat was highest among law enforcement officials (97.1%), followed by transporters (68.8%) and students (9.2%). The majority of khat chewers were in the age range of 16–25 years. The secondary school and the college age (15–25 years) constitute a critical period of lifetime. Adolescence is often a period during which individuals try on new attitudes, roles, and behaviours. Some adolescents choose to engage in risky behaviours. For some, the experience will be one of experimentation, a passing phase. For others, it will be the beginning down a path to problems that follow them into adulthood. There is a fairly consistent pattern that engaging in risky behaviours as a teenager is associated with less successful adult outcomes. In most cases, the earlier one engages in the behaviour, the more likely one faces a bad outcome as an adult. Adolescents seek to develop their own identity, opinions, and values [20]. For adolescents, given the freedom to experiment, this stage also entails taking some risks. When adolescents take risks, the consequences can be negative: car accidents can occur while driving drunk, smoking can lead to cancer, and unprotected sex can lead to unwanted pregnancies and disease. Many factors contribute to the increased vulnerability of adolescents with regard to HIV infection and other risks to their health and well-being that range from biological to social [21]. It is for these reasons that studies of adolescent sexual and other risk-taking behaviour are imperative if we want to reduce the number of sexually-transmitted infections and various risk-taking behaviours amongst adolescents.

Few reports could be found in the literature on the prevalence of khat among the school students. A study in Ethiopia revealed 26.7% life time prevalence rate of khat chewing among students [22]. Another study [15] revealed that the prevalence of khat chewing among secondary school students in south-western Ethiopia was 64.9%. The prevalence rate of current use of khat among medical and paramedical students in north-western Ethiopia [14] was 22.3%.

Milaat et al (2005) reported that current khat prevalence among the general population in Jazan area is 48.7 percent (45.7 percent in rural compared to 61.7 percent in urban areas) [19]. Its use was high in the following provinces: Sabiya (72.5%), Jizan (61.7%), Alhurath (58.1%), Abu Arish (56.8%) and Samtah (55.7%). With improvement in awareness, there is growing evidence that the new generation of students favors the ban on khat even though they continue to chew the leaves before examinations [23]. However, khat prevalence among secondary school and college students in Jazan area was not previously reported. This study was conducted to assess the prevalence and associated risk factors of khat chewing among secondary school and college students in Jazan region. The secondary school and college students were selected, as they represent the future leaders of the community. Their attitude and the way they behave and think will have a great impact on the population.

## Subjects and methods

### Study design

A Cross-sectional Survey was conducted in May 2006 among students (15–25 years old) in Secondary (High) Schools and all Colleges in Jazan region, Southwest of the Kingdom of Saudi Arabia (KSA). Jizan city is the capital of the region and is only 70 km from the Yemen border. The study included the students of Jazan Faculty of Medicine, Jazan Community College, Jazan Engineering and Computer College, Colleges of Teachers (Male and Female) in Jazan, Sabiya, Samta and Farsan, Jazan Female Health Institute and Jazan Health College. The total number of students enrolled in the 11 colleges in 2005–2006 academic year was 18,243 (12,383 females and 5,860 males). The study included also the students of 102 boys' schools in Jizan and Sabiya Education Sectors (with a total number of 25,120 students) and 105 Girls' Schools in Jazan region (with a total number of 21,640 girls).

### Research questions

The aim of this study was to assess the prevalence and risk factors of khat chewing among college and secondary school students in Jazan region.

This study is part of a main research project sought to answer the following research questions:

• What is the prevalence and risk factors of khat chewing among college and secondary school students in Jazan region?

• What is the perceived health and social effects of Khat chewing?

• Whether khat chewing was associated with learning and academic achievement?

• What are the attitudes towards khat chewing and how khat users obtain their supplies of khat

### The sample

The study population includes students at 15–25 years old and excludes those who are outside this range. The study included all the colleges and 20% of the schools. The sample size was 20% of students in the colleges and 20% of the schools of the area. Systematic random sampling technique was applied to select students in each class of the educational institute.

### Instrument

A pre-tested self-administered questionnaire, which was prepared in Arabic, was used for data collection. The independent variables included: class level, residence address (rural versus urban), sex, age, grades, and family history of khat chewing and socioeconomic status of the parents. The main dependent variables were history of khat chewing. The response format is choosing coded answer in the self-administered questionnaire.

The questionnaire was pretested by distribution to the selected students in the classroom from 2 colleges and 4 schools. The instructors allowed the students to complete the questionnaire in the classroom, and collected immediately. The questionnaire were reviewed by the investigators, and modified and updated accordingly.

### Data collection

Ethical clearance and permission was obtained from the local authorities (the local governorment) and Jazan University Deanship of Research. Before the data collection was started permission was also obtained from the Deans of the respective colleges and Directorate of Education Sectors in Jizan and Sabiya. During distribution of the questionnaire, students were informed that the information collected would be kept anonymous and participation was totally voluntary.

The data collection was supervised and coordinated by field supervisors, who were school teachers and faculty members at the colleges. A two-day workshop was conducted at the Faculty of Medicine for training field supervisors. The questionnaires were checked by field supervisors at the end of each day during the survey, for omission of incomplete answers and for coding the responses.

### Data analysis

Data was processed and analyzed using the statistical package for Social Sciences (SPSS) version 11. Descriptive frequencies and Chi-square test was used to test the association between different variables

## Results

Out of the total 10000 questionnaires distributed, 8965 were returned making the response rate 89.65%. The college students participating in the study were 2466 (27.5%) and the secondary schools 6499 (72.5%). The male students were 4639 (51.75%), whereas female students were 4326 (48.25%). About 69.5% of the students were in the age group 15–20 years (Table 1). The mean age of the respondents was 18.9 years (SD = 2.58).

**Table 1:** Demographic Data

**Age Group**	**Colleges**		**Schools**	
	Male (%)	Female (%)	Male (%)	Female (%)
**15–<20**	404 (35.8)	500 (37.7)	2853 (81.3)	2391 (80.6)
**20–25**	724 (64.2)	838 (62.3)	653 (17.3)	579 (18.8)

**Total**	1128 (45.7)	1338 (54.7)	3511 (54)	2988 (46)

**Figure 1:**
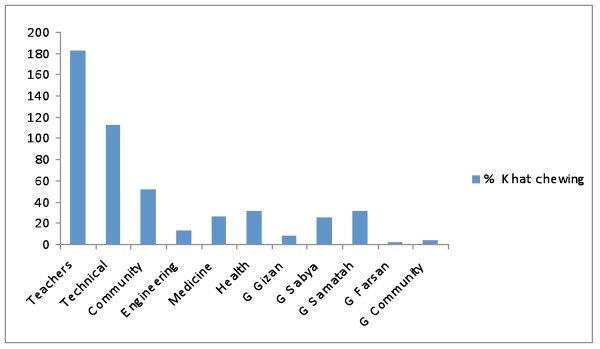
**prevalence of Khat Chewing in Jazan Colleges**. Key: Teacher (Teacher College), Technical (Technical College), Community (Community College), Engineering (Engineering College), Medicine (Faculty of Medicine), Health (College of Health Sciences), G Jizan (Jizan Girls College), G Sabya (Sabya Girls College), G Samatah (Samatah Girls College), G Farasan (Farasan Girls College), G Community (Community Girls College).

The overall prevalence of khat chewing in all the studied population of students was 21.4%. Khat prevalence was high in secondary schools (21.5%) compared to the colleges (15.2%). The life time prevalence rate of Khat chewing in the colleges was: 44.40% in Boys Community College, 43.6% in Boys Technical College, 41.90% in Boys Health College, 38.20% in Engineering and Computer College, 35.80% in Jazan Boys Teachers, 21.40% in Boys College of Medicine, 7.20% in Samtah Girls Education, 4.80% in Sabiya Girls Education, 4.10% in Abu Arish Girls Community College, 3.50% in Jazan Girls Education and 1.40% in Farsan Girls Education (Fig. 1).

The prevalence of khat chewing according to residence, gender, age, and educational sector is represented in Table 2. The prevalence rate of khat chewing in Sabiya educational sector schools (39.20%) was more than that in than in Jizan educational sector schools (18.20%) (p < 0.05) (Table 2). The highest prevalence of Khat chewing was in Fifa province and the lowest was in Farsan province (Table 2).

**Table 2:** Prevalence of Khat Chewing

**Factor**	**Total**	**No of Khat Chewing**	**% Khat Chewing**	**Significance**
**Residence:**				
1. Rural	4901	1003	20.50	p < 0.05
2. Urban	3037	743	24.50	

**Sex:**				
1. Male	4477	1690	37.70	p < 0.05
2. Female	4146	151	3.60	

**Age:**				
1. <15	23	4	17.40	p < 0.05
2. 15–<20	5494	1120	20.40	
3. 20–25	2648	641	24.20	

**Education Sector:**				
1. Jizan	5512	1001	18.20	p < 0.05
2. Sabiya	1118	439	39.20	

There were 151(3.8%) female Khat chewers and 1783 (37.70%) male Khat chewers. Significant difference (p < 0.05) was found between male and female khat chewers (Table 2). Significant difference (p < 0.05) also was found between khat chewers from rural and urban areas. Khat chewers were more in urban areas (24.50%) than in rural areas (20.50%).

Comparing the thirteen provinces showed that khat chewing prevalence differs from one province to another. The highest prevalence noted in Fifa province (63.90%) whereas the lowest prevalence (6.30%) was reported in Farsan province (Table 3).

**Table 3:** Comparison between Khat chewing prevalence among students and general population men (15–25 years) in the different provinces of Jazan Region

**Province**	**Prevalence in students %**	Prevalence in the overall population * (%)	**Significance**
Jizan	10.80	61.7	p < 0.05
Abu Arish	32.90	56.8	p < 0.05
Sabiya	20.30	72.5	p < 0.05
Farasan	6.30	12	p < 0.05
AlAhad	27.10	51.7	p < 0.05
Samatah	24.40	55.7	p < 0.05
AlHurth	34.70	58.1	p < 0.05
Alardah	40.10	38.1	NS
Fifah	63.90	34.7	p < 0.05
Aldaer	48.10	19	p < 0.05
Bish	25.30	20	NS
Damad	31.60	16.5	p < 0.05
Aldarb	9.50	37	p < 0.05

* Quoted after Milaat et al, 2005 [19]

NS = not significant

Table 3 shows a comparison between Khat chewing prevalence among students (findings of the present report) and the overall prevalence in the general population men^19^. Most of the provinces showed that the prevalence of khat chewing among students was significantly lower (p < 0.05) than its overall prevalence in the general population.

## Discussion

It was estimated in a previous survey [19] that the overall prevalence of current Khat use in Jazan region is 48.7 percent. The present study showed that the current prevalence rates of Khat chewing among secondary school and college students was 21.1% and 19.2%, respectively. This means that the prevalence of khat among college and secondary school students is much lower than its prevalence in the general population. The reason for the lower prevalence among secondary school and college students needs further investigation. It could be speculated that the low prevalence of khat among students is due to increased awareness towards the harmful effects of khat in addition to the unavailability of adequate income to purchase khat.

The overall khat use was found in a previous study [19] to be high in the following provinces: Sabiya (72.5%), Jizan (61.7%), Alhurath (58.1%), Abu Arish (56.8%), and Samtah (55.7%). The present study showed that the prevalence of khat use among the students in the same provinces was: Alhurath (34.70%), Abu Arish (32.90%), Samtah (24.40%), Sabiya (20.30%), and Jizan (10.80%). Sixty three per cent of the students surveyed from Faifa province used khat. This shows that among the study participants khat tended to be used more frequently in Faifa than it was in other provinces, with only 6.3 per cent of Farsan respondents using khat. Faifa is a known area for khat production. The Saudi government has enacted a further law prohibiting the expansion of khat cultivation within the Faifa mountain area (near to Jazan city). Khat cultivation is now controlled and supervised by the Ministry of Interior under a local administration called the Faifa Development Authority (established 1978). The authority has offered financial and practical assistance to khat cultivators to develop alternative crops, such as fruit and coffee trees. With the assistance of the National Guards, the authority now monitoring the mountain 24 hours a day and checking people and cars coming from the mountain, in order to detect khat smugglers. However, their control is ineffective in some areas as khat is still used privately in houses of the Faifa Mountain. Visitors from Jazan city and other neighbouring towns can come to the mountain chew khat as they wish. They then leave without taking any khat with them [13].

The rates of prevalence of khat use among students reported in this study are lower (21.4%) compared to similar studies in other countries. A study in Ethiopia revealed 26.7% life time prevalence rate of khat chewing among students [20]. The possible explanations for this difference could be that the Ethiopian study was done only in one college (GCMS). Another study [15] revealed that the prevalence of khat chewing among secondary school students in south-western Ethiopia was 64.9%.

The pattern of use of khat among 479 medical and paramedical students in a boarding college in north-western Ethiopia was studied by an anonymous self-administered questionnaire [11]. The majority of students were males (82.6%) within an average age of 21.2 years. The prevalence rate of current use of khat was 22.3%, which is nearly similar to the prevalence of khat use reported in this study.

A study performed in three towns in south-western Uganda [18] where one hundred and thirty students were compared with thirty five law enforcement officials and sixteen transporters. The study showed that among the students 57 (31.5%) had chewed khat before, 37 (20.4%) still chewing khat. In the three categories of subjects, the use of khat was highest among law enforcement officials (97.1%), followed by transporters (68.8%) and students (9.2%). The majority of khat chewers were in the age range of 16–25 years.

Few reports could be found in the literature on the prevalence of khat among the school students. However, survey studies dealing with other populations were also documented. A study examined the prevalence of khat chewing among women during pregnancy [24]. About 40.7% of the surveyed women reported chewing khat while pregnant during the 5 years before the survey. Another study [25] reported khat use, together with other drugs, among active security personnel and militia in Somalia. It was reported that the most frequent form of drug use is khat chewing (on average, 70.1% in the previous week). In the last cross-sectional assessment of khat intake before the collapse of state of Somalia, Elmi [26] reported that its prevalence in the 1980s in the north of the country was 64% in adult males compared to 21% in the south. It was recently reported in northwestern Somalia (Somaliland) [27] that khat use was more frequent and excessive among male ex-combatants (60%) than among adult male civilian war survivors (28%) and males without war experience (18%; *p *< 0.001). A survey of 1200 adults from a rural Ethiopian community [17] found that the current prevalence of khat chewing was 31.7%. Muslims more than Christians, males more than females, those between the ages 15 and 34 years more than other age groups were habitual users of khat.

The present study revealed that 37.7% of boys and 3.7% of girls are current Khat chewers. Similar differences were reported in a survey carried out in a rural Ethiopian community [16]. It was found that the prevalence of current khat use was 50%. Among current chewers, 17.4% reported taking khat on a daily basis; 16.1% of these were male and 3.4% were female. This higher prevalence of khat use among male respondents is in accordance with the greater cultural acceptance in a Moslem society of men rather than women using it. One limitation of this study is that 100% response was not obtained. This is usually one of the limitations of self-administered questionnaires [28]. The other limitation could be that all students might not give genuine answer to the questions. This might underestimate the prevalence of khat chewing in this study.

A previous survey [19] estimated that the highest overall prevalence of khat use in Jazan region was reported in rural areas (61.7 percent) compared to urban areas (45.7 percent). A survey carried out in a rural Ethiopian community [16] on a total of 10,468 adults found that more than half of the study population (55.7%) reported lifetime khat chewing experience and the prevalence of current use was 50%. The findings of the present survey showed that Khat chewers among students were more in urban areas (24.50) than in rural areas (20.50%), this difference, however, was not statistically significant.

The secondary school and the university age (15–25 years) constitute a critical period of lifetime. As in previous studies [29] the present study revealed that the prevalence of khat chewing increases with age and year of study. In a study that involved all the instructors in four colleges in north-west Ethiopia [30], it was found that the current prevalence rate of khat chewing was 21.0%. The majority of the instructors (40.0%) started khat chewing while they were senior high school or first year college students [30]. The main reasons mentioned for starting chewing were "peer pressure" and "for relieving stress". This is an important indication to direct interventions towards decreasing the prevalence of these habits. Additionally, students need counselling service on ways of coping with their problems.

Several studies revealed also that it is during the secondary school and the college age (15–25 years) that khat use is associated with risk behaviours. This could be attributed to biological, psychological, sociocultural and economic factors. It was found that the young people in Ethiopia [31], particularly those aged 15–25 years, are generally at a high risk of HIV/AIDS and other reproductive health problems. Of the 628 study subjects, 64.8% had experienced sexual intercourse at the time of the survey. In another study [30] a probabilistic national sample of 20,434 in-school and out-of-school Ethiopian youths aged between 15 and 24 years of age were interviewed regarding khat use. It was found that daily Khat intake was associated with unprotected sex.

## Conclusion

Based on the findings of the present study, it is suggested that measurements should be arranged for raising awareness of the students, in addition to other measurements such as; application of deterrent laws, prohibition of cultivation of khat, and border control by advanced technologies. Strict law enforcement should be applied to dry the region by destruction of khat trees and ban imports of khat from Yemen. Disseminating health education awareness could be done through media like television and newspapers, arranging religious programs like lectures in mosques and establishing Khat Quit Clinics.

## Competing interests

The author declares that they have no competing interests.

## Authors' contributions

I am the principal investigator, designed the study, had full responsibility for its overall management drafted and revised the article.

## Authors' informations

The author is currently the Dean of the Faculty of Medicine, Jazan University, Jazan, Saudi Arabia. Moreover he is the Head of Gastroenterology Unit, King Fahd Central Hospital, Jazan. Dr. Ageely is Member of the American College of Gastroenterology, Saudi Gastroenterology Association and Saudi Medical Education Society.
